# Determinants of HIV Testing Uptake Among People Who Use New Psychoactive Substances in Kazakhstan: A Multi-Regional Cross-Sectional Study

**DOI:** 10.3390/healthcare14091183

**Published:** 2026-04-28

**Authors:** Roza Kuanyshbekova, Venera Baisugurova, Gulzar Shah, Bushra Shah, Gulshara Aimbetova, Manshuk Ramazanova, Indira Karibayeva, Nargiza Yussupova, Botagoz Turdaliyeva

**Affiliations:** 1 Department of Science, Kazakh Scientific Center of Dermatology and Infectious Diseases, Almaty 050002, Kazakhstan; r.kuanyshbekova@kncdiz.kz (R.K.); ns.yussupova@kncdiz.kz (N.Y.); 2 Department of Biostatistics and Foundations of Scientific Research, Asfendiyarov Kazakh National Medical University, Almaty 050012, Kazakhstan; baysugurova.v@kaznmu.kz; 3 Department of Health Policy and Community Health, Jiann-Ping Hsu College of Public Health, Georgia Southern University, Statesboro, GA 30460, USA; bs06779@georgiasouthern.edu (B.S.); ik01379@georgiasouthern.edu (I.K.); 4 Department of Public Health, Asfendiyarov Kazakh National Medical University, Almaty 050012, Kazakhstan; agulshara18@gmail.com (G.A.); manramazanova@gmail.com (M.R.); 5 Department of Public Health and Social Sciences, Kazakhstan Medical University “KSPH”, Almaty 050060, Kazakhstan; 6 Department of Research Management, JSC Research Institute of Cardiology and Internal Diseases, Almaty 050012, Kazakhstan; 7 Department of Nursing, Asfendiyarov Kazakh National Medical University, Almaty 050012, Kazakhstan

**Keywords:** new psychoactive substances, HIV testing, people who use drugs, psychostimulant injection, HIV prevention, Kazakhstan, structural determinants

## Abstract

**Highlights:**

**What are the main findings?**

- HIV testing uptake among adults who use new psychoactive substances (NPS) in Kazakhstan was high (86.7%), but varied by age, education, peer communication, and substance use patterns.
- Engagement with HIV prevention services, use of prevention centers, injectable psychostimulant use, and discussion of HIV within peer networks were significantly associated with the likelihood of HIV testing.

**What are the implications of the main findings?**

- Integrating HIV testing with prevention services may help sustain or expand testing uptake among people who use NPS. Accordingly, these associations should not be interpreted as evidence that engagement with prevention services independently increases HIV testing uptake.
- Strengthening peer-based communication and outreach strategies could enhance HIV testing uptake and support earlier diagnosis in emerging substance-using populations.

**Abstract:**

Background: New psychoactive substances (NPS) represent an evolving component of global substance use patterns and may contribute to HIV transmission through both injection-related and sexual risk behaviors. In Kazakhstan, where HIV incidence has increasingly shifted toward sexual transmission, evidence on HIV testing among NPS users remains limited. This study examined behavioral, social, and structural factors associated with HIV testing in this population. Methods: A cross-sectional study was conducted among 1500 adults reporting NPS use across six regions of Kazakhstan. Data were collected using structured interviewer-administered questionnaires. The primary outcome was self-reported HIV testing (ever tested: yes/no). Independent variables included sociodemographic characteristics, substance use behaviors, sexual practices, peer communication about HIV, and structural access to prevention services. Univariable logistic regression with Bonferroni correction (*p* < 0.001) was used for variable screening. Multivariable logistic regression models estimated adjusted odds ratios (AORs) with 95% confidence intervals (CIs). Model discrimination was assessed using the area under the receiver operating characteristic curve (AUC). Results: Overall, 86.7% of participants reported prior lifetime HIV testing. In the multivariable model (*n* = 1482), older age was associated with higher odds of testing (AOR 1.06 per year; 95% CI 1.04–1.08; *p* < 0.001). Compared with participants holding a bachelor’s degree or higher, those without a high school diploma had lower odds of testing (AOR 0.50; 95% CI 0.28–0.89). Injectable psychostimulant use was also associated with testing (AOR 1.40; 95% CI 1.21–2.01). Participants who never discussed HIV within peer networks were less likely to have been tested (AOR 0.69; 95% CI 0.49–0.97). Engagement with HIV prevention services (AOR 0.54; 95% CI 0.39–0.75) and use of prevention centers (AOR 0.63; 95% CI 0.45–0.87) were significantly associated with testing. The model demonstrated acceptable discrimination (AUC = 0.725). Conclusions: Lifetime HIV testing uptake among NPS users in Kazakhstan is high but influenced by educational attainment, peer communication, injection practices, and engagement with prevention services. Strengthening integration of prevention services and expanding peer-based outreach may improve equitable access to HIV testing in this population.

## 1. Introduction

New psychoactive substances (NPS) represent a heterogeneous and rapidly evolving group of synthetic compounds designed to mimic the effects of traditional controlled drugs while often evading legal regulation [1]. Meta-analytic estimates suggest that synthetic cannabinoids, phencyclidine-type compounds, and synthetic cathinones represent the most prevalent NPS classes worldwide, with lifetime use generally ranging from approximately 7% to 15% [2]. Unlike established substances such as heroin or cannabis, NPS are characterized by shifting chemical formulations, variable potency, and limited evidence regarding toxicity and long-term health consequences [3,4]. These substances include synthetic cathinones, cannabinoids, and other stimulants that may be consumed through multiple routes, including injection, inhalation, intranasal (snorted), and oral administration [5]. The rapid proliferation of NPS has posed significant challenges to surveillance systems, harm reduction services, and public health responses due to their dynamic composition and patterns of use.

In Kazakhstan and across Eastern Europe and Central Asia, substance use remains a key structural driver of the HIV epidemic. Historically, injection opioid use has been the dominant risk factor with suboptimal viral suppression levels among people who inject drugs [6], but recent years have seen diversification in substance use patterns, including increased availability and consumption of psychostimulant-type NPS [7,8]. Stimulant use—particularly when injected or used in sexualized contexts—has been associated with high-risk sexual behaviors, condomless intercourse, multiple partnerships, and transactional sex [9]. These dynamics are particularly relevant among key populations, including men who have sex with men (MSM), where chemsex and stimulant use have been linked to rising HIV and other sexually transmitted infection (STI) incidence [10]. In Kazakhstan, surveillance data indicate a growing proportion of new HIV cases attributable to sexual transmission, including among MSM, suggesting that intersections between substance use and sexual risk warrant closer examination [11,12].

Globally, NPS use has expanded substantially over the past decade. By late 2024, more than 980 NPS were under surveillance in Europe, with 26 substances newly detected in 2023 [13]. Although precise prevalence data remain limited due to underreporting and evolving drug classifications, regional monitoring indicates increasing stimulant-type NPS use in urban centers of Central Asia [14]. In Kazakhstan, harm reduction services report growing engagement with individuals who use synthetic stimulants and mixed substances, often in conjunction with existing opioid use [7,15]. However, epidemiologic data specific to NPS users remain sparse, and the extent to which this population is engaged in HIV prevention and testing services has not been systematically characterized.

HIV testing is a cornerstone of effective HIV prevention and care, enabling early diagnosis, timely initiation of antiretroviral therapy, and interruption of onward transmission. Despite the expansion of HIV testing services in Kazakhstan, including community-based and prevention center-based models, gaps in testing uptake persist among people who use drugs [16,17]. While numerous studies have examined HIV testing among traditional opioid users, far less is known about determinants of testing among NPS users, whose risk profiles may differ due to stimulant-associated sexual risk behaviors, episodic injection practices, and distinct social networks. Understanding the behavioral, social, and structural factors associated with HIV testing in this emerging population is critical for tailoring prevention strategies and improving service linkage.

This study is guided by the Health Belief Model (HBM), focusing on the constructs of perceived risk and perceived susceptibility [18]. According to the HBM, individuals’ engagement in preventive health behaviors, such as HIV testing, is shaped by their beliefs about personal vulnerability to disease and the severity of potential health outcomes [19]. Among people who use NPS, perceived susceptibility to HIV may be heightened by awareness of sexual risk behaviors and injection practices. In contrast, perceived risk reflects recognition of the broader consequences of HIV infection, including stigma, morbidity, and mortality. By applying this framework, the study examines how these perceptions, alongside sociodemographic and structural factors, influence HIV testing uptake.

Addressing this gap, the present study examines correlates of HIV testing uptake among people who use NPS across six regions of Kazakhstan. By identifying sociodemographic, behavioral, and structural factors independently associated with testing—through the lens of perceived risk and susceptibility—the study aims to inform targeted HIV prevention programming and optimize service delivery models for populations affected by evolving patterns of substance use.

## 2. Materials and Methods

### 2.1. Study Design and Setting

We conducted a multi-regional cross-sectional study among people who use NPS in Kazakhstan to examine factors associated with HIV testing uptake. Data were collected across six regions (Astana, Karaganda, Kostanay, Oskemen, Petropavlovsk, and Shymkent), representing diverse geographic and service-delivery contexts within the country. Participants were recruited through community outreach, harm reduction programs, and HIV prevention centers using structured, interviewer-administered questionnaires. Recruitment and data collection were conducted by trained outreach workers who have established access to and trust within hard-to-reach key populations. NPS users were contacted through these outreach networks, and interviews were administered directly by outreach workers responsible for participant recruitment and data collection. Eligible participants were aged 18 years or older and reported use of NPS or related psychoactive substances within the preceding 12 months. All participants provided written informed consent prior to participation. Individuals unable to provide consent were excluded.

A total of 1500 individuals were enrolled. After excluding observations with incomplete data on variables included in the multivariable model (less than 95%), 1482 participants comprised the complete-case analytic sample. The target sample size was determined to ensure adequate statistical power to detect modest associations between behavioral and structural exposures and HIV testing uptake. Assuming an HIV testing prevalence of approximately 20% in the study population, a two-sided α of 0.05, and 80% power, a sample of 1500 participants provides sufficient precision to detect odds ratios in the range of 1.3–1.4 for binary exposures with moderate prevalence. Sample size was estimated using the RiskCalc.org calculator for cross-sectional surveys with a proportional outcome [20].

The study was conducted in accordance with established ethical standards for human-subject research. All participants provided written informed consent before taking part. Ethical approval was granted by the Local Ethics Committee of Kazakhstan’s Medical University “KSPH” (IRB-134-2024; 19 December 2024). To ensure confidentiality, no personally identifiable information was collected, and all data were anonymized through assignment of unique codes to each questionnaire. Data security was maintained through restricted access and secure storage on password-protected computers. Interviews were conducted in private settings to minimize disclosure risks. In accordance with the approved study protocol, compensation was provided to outreach workers conducting the survey; participants did not receive direct financial compensation.

### 2.2. Measures

The primary outcome was self-reported HIV testing uptake, assessed as a binary variable indicating whether the participant had ever undergone HIV testing (Yes/No). “Ever tested” was defined as having undergone HIV testing at least once at any time prior to the survey.

Independent variables were selected a priori based on established behavioral and structural frameworks relevant to HIV risk and service utilization among people who use drugs. Sociodemographic characteristics included age (continuous, in years), gender, marital status (divorced/widowed, married, not married), education level (Bachelor’s degree or higher, did not complete high school, high school, some technical education), region of residence (Astana, Karaganda, Kostanay, Oskemen, Petropavlovsk, and Shymkent) and monthly income level (≤100,000 KZT, 100,000–200,000 KZT, ≥200,000 KZT).

Substance use behaviors were assessed using self-reported measures capturing recent and lifetime patterns of drug use and related risk contexts. Participants reported use of marijuana, heroin (injection and inhalation), and methadone (injection and per-oral), as well as psychostimulants (injection and inhalation), including commonly reported substances such as synthetic cathinones (e.g., mephedrone, α-PVP), amphetamine-type stimulants, and other stimulant-type new psychoactive substances reported in the regional context. Polysubstance use was characterized by reported use of drug mixtures via injection and via inhalation or oral routes. Injection-related risk was measured by unsafe injection practices, categorized as never versus ever, and by reporting needle use after a person known to be HIV-positive. Social and contextual risk environments were evaluated by the frequency of HIV-related discussions among users (never, sometimes, often, or unknown), chemsex use, and perceptions of whether sexual activity under the influence of drugs increases the risk of sexually transmitted infections and HIV.

Sexual behavior variables captured engagement in transactional sex and patterns of sexual risk within the preceding three months. Participants reported whether they had exchanged sex for money or drugs and whether they had exchanged sex for food or accommodation. Condom use during paid sex was assessed and categorized as always, sometimes, or never. Sexual network size was measured by the number of sexual partners in the past three months and dichotomized as two or fewer versus three or more partners. Participants also reported engagement in anal sex with an irregular partner during the same period.

Structural access to services was evaluated through self-reported receipt of HIV prevention services and usual sources of non-substance-related medical care, including outpatient public clinics, hospitals, outpatient private clinics, HIV prevention centers, and non-governmental organizations. Perceived barriers to safe injection practices were assessed by asking participants what prevented them from avoiding shared needles or equipment, with response options including withdrawal symptoms, fear of being caught, lack of opportunity to obtain a syringe, financial constraints, loss of control, absence of fear of HIV, or reporting no barriers. Similarly, barriers to consistent condom use were assessed through reported reasons such as loss of control, absence of HIV fear, lack of opportunity to obtain condoms, financial constraints, fear of expressing concern, partner refusal of condom use, no barriers, or other reasons.

Consistent with the HBM, selected variables were mapped to key constructs. Perceived susceptibility was approximated by engagement in high-risk behaviors (e.g., injection drug use, multiple sexual partners), which may reflect individuals’ increased awareness of their vulnerability to HIV infection, while perceived risk and severity were reflected in awareness-related variables such as HIV-related discussions within peer networks, which may shape perceptions of the consequences and likelihood of infection. Structural variables, including engagement with prevention services and access to HIV prevention centers, were conceptualized as cues to action that facilitate testing behavior.

### 2.3. Statistical Analysis

Descriptive statistics were computed as means with standard deviations for continuous variables and frequencies with percentages for categorical variables. Differences by HIV testing status were assessed using Wilcoxon rank-sum tests for continuous variables and Pearson’s χ^2^ or Fisher’s exact tests for categorical variables, as appropriate.

Univariable logistic regression models were fitted to estimate crude odds ratios (ORs) and 95% confidence intervals (CIs) for the association between each independent variable and HIV testing uptake. To account for multiple comparisons across candidate predictors, a conservative Bonferroni correction was initially applied (*p* < 0.001) for variable screening. Given the potential for over-conservatism, sensitivity analyses using alternative correction approaches (Holm–Bonferroni) were conducted to ensure robustness of variable selection.

Multivariable logistic regression was subsequently performed to estimate adjusted odds ratios (AORs) and 95% CIs for factors independently associated with HIV testing uptake. Model discrimination was evaluated using the area under the receiver operating characteristic curve (AUC), and overall model fit was assessed using the Akaike Information Criterion (AIC) and McFadden’s pseudo-R^2^. All statistical tests were two-sided, and statistical significance in multivariable analyses was defined as *p* < 0.05. All analyses were conducted using R (version 4.5.2; R Foundation for Statistical Computing, Vienna, Austria) within RStudio (version 2025.09.2+418; Posit Software, PBC, Boston, MA, USA) [21,22]. Given the established role of sexual risk behaviors in HIV transmission among stimulant users, additional exploratory analyses were conducted including sexual behavior variables (e.g., transactional sex, condom use, number of sexual partners, and anal sex) to assess their associations with HIV testing. These results are presented in Supplementary Table S4.

## 3. Results

**Figure 1:**
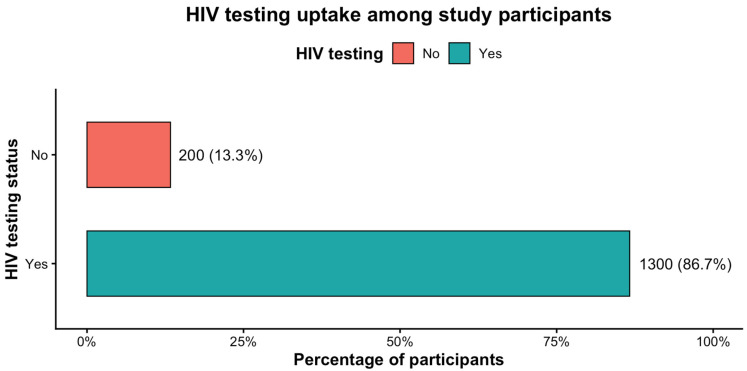
Lifetime HIV testing uptake among study participants.

As shown in Figure 1, the majority of participants reported having undergone HIV testing. Of the 1500 individuals enrolled, approximately 1300 (86.7%) indicated prior HIV testing, whereas 200 (13.3%) reported never having been tested. This high level of reported lifetime testing uptake suggests substantial engagement with HIV screening services among people who use NPS across the six participating regions.

Table 1 summarizes participant characteristics stratified by HIV testing status. Individuals who reported prior HIV testing were significantly older than those who had never been tested (mean 37 years [SD 9] vs. 33 years [SD 10], *p* < 0.001). Gender distribution was comparable across groups, with men comprising the majority in both strata (79% among those never tested vs. 80% among those tested; *p* = 0.7). Marital status differed significantly by testing status (*p* = 0.001): participants who had been tested were more frequently divorced or widowed (31% vs. 20%), whereas those who had never been tested were more often not married (71% vs. 57%). Educational attainment also varied (*p* < 0.001), with a higher proportion of “some technical” education among those tested (45% vs. 28%) and a higher proportion of incomplete high school among those never tested (23% vs. 10%); the proportion with bachelor’s degree or higher was modest in both groups but lower among those tested (8.8% vs. 14%). Residency (urban vs. rural) and employment status did not differ significantly between groups (*p* = 0.3 for both), reflecting broadly similar structural profiles with predominantly urban residence in the sample. Income distribution showed a borderline difference (*p* = 0.076), with a greater share of participants earning ≥200,000 KZT among those tested (38% vs. 30%). Finally, HIV testing uptake varied significantly by region (*p* < 0.001), indicating meaningful geographic heterogeneity in testing patterns across the six study sites. Further details on participant characteristics, including substance use, sexual behaviors, and structural access factors stratified by HIV testing status, are presented in Supplementary Table S1.

**Figure 2:**
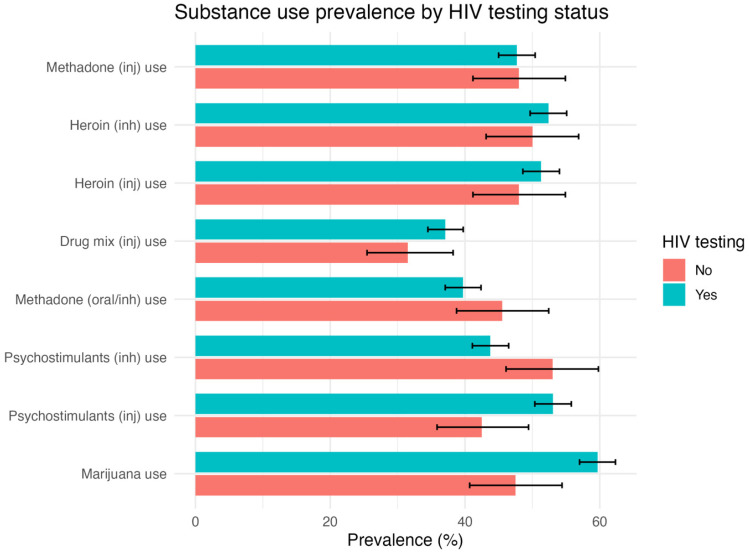
Prevalence of substance use by HIV testing status among NPS users in Kazakhstan.

Figure 2 illustrates the prevalence of selected substance use behaviors stratified by HIV testing status. Compared with participants who had never undergone HIV testing, those who reported prior testing demonstrated higher prevalence of marijuana use (60% vs. 48%; *p* = 0.001) and psychostimulant injection use (53% vs. 43%; *p* = 0.005). Psychostimulant inhalation use also differed significantly between groups (44% among those tested vs. 53% among those untested; *p* = 0.015), although the direction of association varied across stimulant routes of administration. No statistically significant differences were observed for heroin use (injection or inhalation), methadone use (injection or oral), or mixed substance use patterns.

Table 2 presents the multivariable logistic regression results identifying factors independently associated with HIV testing. Older age was consistently associated with higher odds of having been tested (AOR 1.06 per year, 95% CI 1.04–1.08; *p* < 0.001). Education showed independent associations after adjustment: compared with participants with a bachelor’s degree or higher, those who did not complete high school had lower odds of HIV testing (AOR 0.50, 95% CI 0.28–0.89; *p* = 0.020). Social norms and peer communication were also relevant; participants reporting never discussing HIV among users had lower odds of testing (AOR 0.69, 95% CI 0.49–0.97; *p* = 0.034) compared with those who sometimes discussed HIV, while frequent discussions were not significantly different (*p* = 0.368). Participants who reported injectable psychostimulant use had significantly higher odds of testing (AOR 1.40, 95% CI 1.21–2.01; *p* = 0.003) compared with non-users. Structural service indicators remained strongly associated with testing: absence of engagement with HIV prevention services (AOR 0.54, 95% CI 0.39–0.75; *p* < 0.001) and not receiving care at HIV prevention centers as a usual source of care (AOR 0.63, 95% CI 0.45–0.87; *p* = 0.005) were both independently associated with HIV testing. In contrast, marital status, marijuana use, and perceived loss of control as a barrier to condom use were not statistically significant in the adjusted model. Univariable logistic regression results used for variable screening are provided in Supplementary Table S2. Sensitivity analyses using Holm–Bonferroni correction yielded comparable variable selection, supporting the robustness of the final multivariable model.

The final multivariable model included variables selected based on both univariable screening and a priori conceptual relevance. The final model showed acceptable discriminatory performance (AUC = 0.725) and modest explanatory capacity (McFadden R^2^ = 0.109). Full model fit statistics are provided in Supplementary Table S3.

Inclusion of sexual behavior variables did not materially alter the direction or magnitude of the main associations. Sensitivity analyses restricted to subgroups with lower engagement in prevention services and to urban participants yielded broadly similar patterns, although estimates were less stable due to reduced sample size. The reduced sample size in supplementary models resulted in wide confidence intervals and unstable estimates, limiting the interpretability of subgroup analyses (Supplementary Table S4).

## 4. Discussion

This study found that lifetime HIV testing uptake among people who use NPS in Kazakhstan was relatively high. Our findings highlight both individual and structural determinants of HIV testing among NPS users, suggesting that prevention services are reaching a substantial proportion of this population but that educational and social disparities remain.

The observed high testing uptake contrasts with prior studies among opioid users in Kazakhstan and other parts of Central Asia, where engagement with HIV testing services has often been lower. This difference may reflect both the expansion of community-based testing initiatives in recent years and the distinct risk profiles of stimulant-type NPS users, who face heightened sexual transmission risks and may therefore be more motivated to test. Low-threshold HIV testing and harm reduction approaches, such as directly assisted self-testing and social network-based case finding, remain critical for expanding prevention reach and improving access to care among key populations in Kazakhstan [23]. International evidence similarly indicates that stimulant use, particularly in sexualized contexts, is associated with higher HIV testing rates compared to opioid use. However, persistent gaps in linkage to care and prevention services highlight ongoing challenges [9,24]. Moreover, the observed association between education and testing uptake aligns with global findings that higher educational attainment enhances health literacy and service utilization. At the same time, the influence of peer communication echoes social network studies emphasizing the role of collective norms in shaping testing behaviors [25,26]. Importantly, the observed associations between engagement with HIV prevention services and testing uptake likely reflect reverse causality or service-linked testing, whereby testing is delivered as part of service contact rather than representing an independent protective behavioral effect. Therefore, these associations should be interpreted with caution.

Recent advances in surveillance have further improved understanding of NPS use and its associated harms through integrated approaches, including toxicological testing in emergency departments, sentinel population surveys, drug checking programs, syringe service data, wastewater-based epidemiology, and retrospective clinical analyses [27,28]. The harmonization of these complementary data streams provides a more comprehensive picture of evolving drug markets and exposure patterns. However, surveillance alone is insufficient; translating these insights into equitable, stigma-free prevention services is essential to ensure that emerging substance use trends do not exacerbate HIV transmission dynamics in Kazakhstan and the broader region.

The political environment in Kazakhstan presents significant challenges for sustaining and expanding HIV prevention among key populations. Recent criminalization of same-sex sexual behavior propaganda [29] and heightened stigma against MSM [30] have created barriers to accessing HIV testing and prevention services, including pre-exposure prophylaxis (PrEP). These legal changes risk driving MSM and other marginalized groups underground, reducing their willingness to engage with health services due to fear of surveillance, discrimination, or prosecution. For NPS users who overlap with MSM networks, particularly in the context of increasing chemsex practices involving stimulant-type substances [31], this criminalization may undermine the very gains observed in testing uptake, as structural stigma erodes trust in prevention centers and community-based programs. Chemsex environments, often characterized by multiple partners, prolonged sexual sessions, and polysubstance use, may further amplify HIV transmission risk while simultaneously heightening vulnerability to policing and discrimination [32,33,34,35]. The findings of this study must therefore be interpreted within a broader socio-political context in which legal and policy developments may reverse progress in HIV prevention, despite strong engagement with testing and prevention services among NPS users.

The findings can be interpreted within the framework of the HBM. Engagement with HIV prevention services and exposure to HIV-related discussions among peers may function as cues to action, facilitating testing behavior. In contrast, factors such as loss of control in sexual situations may reflect perceived barriers that hinder preventive behaviors. These patterns suggest that both structural engagement and individual-level perceptions play a role in shaping HIV testing uptake among NPS users. This conceptual framing supports the relevance of multi-level interventions targeting both individual perceptions and structural access to services.

Several limitations should be acknowledged. First, the reliance on self-reported HIV testing introduces potential recall and social desirability bias, particularly in a stigmatized environment. Second, the cross-sectional design precludes causal inference regarding the associations observed between education, peer communication, and service engagement with HIV testing. Third, the sample was predominantly urban, limiting generalizability to rural populations where access to HIV testing and prevention services is often more constrained and where structural barriers may be substantially different. Fourth, while the study captured substance use behaviors, it did not fully explore sexual risk practices, which are critical for understanding HIV vulnerability among NPS users. Fifth, the recruitment strategy may have introduced selection bias. Participants were primarily recruited through harm reduction programs and HIV prevention centers, which likely oversampled individuals already engaged with health services. This may partially explain the relatively high HIV testing uptake observed in the study, potentially overestimating testing coverage among the broader population of NPS users, particularly those not connected to services. Additionally, the current socio-political environment may contribute to underrepresentation of certain high-risk subgroups. Heightened stigma, legal constraints, and fear of disclosure—particularly among individuals engaged in same-sex behavior or chemsex practices—may have reduced participation or led to underreporting of sensitive behaviors. As a result, the study may underestimate both risk behaviors and unmet needs for HIV testing within more hidden populations. The use of “ever tested” as the outcome measure does not capture the frequency or recency of HIV testing and may therefore overestimate current engagement with testing services. Finally, the rapidly evolving legal and political context in Kazakhstan may affect service utilization in ways not captured during the study period, particularly among MSM and other criminalized populations.

## 5. Conclusions

This study demonstrates that lifetime HIV testing uptake among people who use NPS in Kazakhstan is relatively high, driven by age, education, peer communication, and engagement with prevention services. However, disparities remain among younger and less educated individuals, and structural stigma threatens to undermine service access for marginalized groups. By situating these findings within the Health Belief Model framework, where perceived susceptibility and risk shape testing behaviors, the study underscores the need for tailored interventions that strengthen peer communication, expand educational outreach, and safeguard prevention services against political and legal barriers. Ultimately, the results support the study’s aim of informing targeted HIV prevention programming and optimizing service delivery models for populations affected by evolving patterns of substance use in Kazakhstan.

## Figures and Tables

**Table 1:** Sample characteristics by HIV testing status.

Characteristic	No N = 200 ^1^	Yes N = 1300 ^1^	*p*-Value ^2^
Age	33 (10)	37 (9)	<0.001
Gender			0.7
Female	42 (21%)	256 (20%)	
Male	158 (79%)	1044 (80%)	
Marital			0.001
Divorced/widowed	39 (20%)	408 (31%)	
Married	20 (10%)	145 (11%)	
Not married	141 (71%)	747 (57%)	
Education			<0.001
Bachelor and higher	27 (14%)	114 (8.8%)	
Did not complete high school	45 (23%)	135 (10%)	
High school	72 (36%)	470 (36%)	
Some technical	56 (28%)	581 (45%)	
Residency			
Urban	195 (98%)	1226 (94%)	0.3
Rural	5 (2.5%)	74 (6%)	
Employment			0.3
Unemployed	59 (30%)	329 (25%)	
Full time	72 (36%)	537 (41%)	
Part time	69 (35%)	434 (33%)	
Income level (KZT)			0.076
100,000–200,000	60 (30%)	350 (27%)	
200,000 and above	60 (30%)	488 (38%)	
100,000 or below	80 (40%)	462 (35%)	
Region			<0.001
Astana	37 (19%)	213 (16%)	
Karaganda	45 (23%)	205 (16%)	
Kostanay	19 (9.5%)	231 (18%)	
Oskemen	32 (16%)	218 (17%)	
Petropavlovsk	49 (25%)	201 (15%)	
Shymkent	18 (9.0%)	232 (18%)	

^1^ Mean (SD); n (%). ^2^ Wilcoxon rank sum test; NA; Pearson’s Chi-squared test; Fisher’s exact test.

**Table 2:** Multivariable Logistic Regression: Factors Associated with HIV Testing.

	Variable	Adjusted OR (95% CI)	*p*-Value
Age	Age	1.06 (1.04, 1.08)	<0.001
Marital (Ref. Divorced/widowed)	Married	0.88 (0.48, 1.65)	0.691
	Not married	0.77 (0.50, 1.16)	0.225
Education (Ref. Bachelor and higher)	Did not complete high school	0.50 (0.28, 0.89)	0.020
	High school	1.12 (0.65, 1.86)	0.682
	Some technical	1.90 (0.91, 3.19)	0.170
HIV talk among users	Never	0.69 (0.49, 0.97)	0.034
	Often	1.28 (0.76, 2.25)	0.368
Psychostim (inj) (Ref. No)	Yes	1.40 (1.21, 2.01)	0.003
Marijuana (Ref. No)	Yes	1.31 (0.92, 1.87)	0.138
Received HIV prevention services (Ref. Yes)	No	0.54 (0.39, 0.75)	<0.001
HIV prevention centers (Ref. Yes)	No	0.63 (0.45, 0.87)	0.005
What prevents you from always using condoms?			
Loss of control (Ref. No)	Yes	0.77 (0.54, 1.11)	0.162

## Data Availability

The datasets presented in this article are not readily available because the HIV-related data used in this study contain sensitive information and were collected exclusively for the purposes of this research under institutional ethics approval. In accordance with IRB requirements, the data will be securely retained and destroyed 3 years after publication of the authorized research articles. Access to the data is restricted; however, reasonable and well-justified requests may be considered by the corresponding author, subject to approval by the local ethics committee of Kazakhstan’s Medical University. Requests to access the datasets should be directed to BT: turdaliyeva@kncdiz.kz.

## Supplementary Materials

The following supporting information can be downloaded at https://www.mdpi.com/article/10.3390/healthcare14091183/s1. Table S1. Sample characteristics by HIV testing status. Table S2. Univariable logistic regression: HIV testing (Bonferroni selection). Table S3. Model Fit Statistics. Table S4. Supplementary Sensitivity Analyses.
