# Supplementary material for: Determinants of HIV Testing Uptake Among People Who Use New Psychoactive Substances in Kazakhstan: A Multi-Regional Cross-Sectional Study
Source: Healthcare (Basel). 2026 Apr 28;14(9):1183. doi: 10.3390/healthcare14091183 (PMC13163755; doi:10.3390/healthcare14091183)
Supplement: Supplementary file 1 [file healthcare-14-01183-s001.zip › healthcare-4255354-supplementary.pdf]

Table S1. Sample characteristics by HIV testing status

**Characteristic**	**No**<br>N = 200[1]	**Yes**<br>N = 1,300[1]	**p-value**[2]
Substance use behavior			
Marijuana use	95 (48%)	776 (60%)	0.001
Heroin (inj) use	96 (48%)	667 (51%)	0.4
Heroin (inh) use	100 (50%)	681 (52%)	0.5
Metadon (inj) use	96 (48%)	620 (48%)	>0.9
Metadon (per oral) use	91 (46%)	516 (40%)	0.12
Psychostim (inj) use	85 (43%)	690 (53%)	0.005
Psychostim (inh) use	106 (53%)	569 (44%)	0.015
Mix (inj) use	63 (32%)	482 (37%)	0.13
Mix (inh or per oral) use	92 (46%)	679 (52%)	0.10
Unsafe injection use			0.4
Never	171 (86%)	1,047 (81%)	0.0946
Ever	29 (14.5%)	253 (19.5%)	
Needle use after an HIV positive person	103 (52%)	623 (48%)	0.3
HIV talk among users			0.002
Sometimes	66 (33%)	567 (44%)	
Never	111 (56%)	544 (42%)	
Often	23 (12%)	186 (14%)	
Unknown	0	3	
Chemsex use	108 (54%)	744 (57%)	0.4
Do you think sexual activity under the influence of drugs increases the risk of sexually transmitted infections (STIs) and HIV?	82 (41%)	551 (42%)	0.7
Sexual behavior			
Paid sex for money and drugs	92 (46%)	712 (55%)	0.021
Paid sex for food and stay	86 (43%)	657 (51%)	0.047
Condom use for paid sex			0.269

Characteristic	No<br>N = 200[1]	Yes<br>N = 1,300[1]	p-value[2]
Always	40 (25%)	223 (23%)	
Sometimes	54 (34.0%)	294 (29.7%)	
Never	65 (41%)	473 (48%)	
Number of sexual partners (past 3 months)			0.0802
2 or less	118 (62.1%)	832 (69.2%)	
3 and more	72 (37.9%)	371 (30.8%)	
Anal sex with an irregular partner (past 3 months)	94 (47%)	690 (53%)	0.11
Structural access to services			
Received HIV prevention services	122 (61%)	535 (41%)	<0.001
Where do you usually go to receive a medical treatment (not related to a substance use)			
Outpatient public clinic	68 (34%)	506 (39%)	0.2
Hospital	90 (45%)	604 (46%)	0.7
Outpatient private clinic	90 (45%)	620 (48%)	0.5
HIV prevention centers	92 (46%)	465 (36%)	0.005
NGO	83 (42%)	610 (47%)	0.2
What prevents you from injecting drugs safely (e.g., avoiding shared needles or equipment)?			
Withdrawal symptoms	71 (36%)	416 (32%)	0.3
Danger of being caught	90 (45%)	571 (44%)	0.8
Absence of the opportunity to buy a syringe	97 (49%)	643 (49%)	0.8
Lack of money	93 (47%)	609 (47%)	>0.9
Loss of control	71 (36%)	428 (33%)	0.5
Absence of HIV fear	88 (44%)	557 (43%)	0.8
Nothing	104 (52%)	724 (56%)	0.3
What prevents you from always using condoms?			
Loss of control2	60 (30%)	522 (40%)	0.006
Absence of HIV fear	103 (52%)	607 (47%)	0.2
Absence of the opportunity to buy a condom	93 (47%)	656 (50%)	0.3

Characteristic	No<br>N = 200[1]	Yes<br>N = 1,300[1]	p-value[2]
Lack of money for a condom	88 (44%)	635 (49%)	0.2
Afraid to show fear	80 (40%)	572 (44%)	0.3
Partner refusal of condom use	70 (35%)	532 (41%)	0.11
Nothing	131 (66%)	742 (57%)	0.025
Other	96 (48%)	668 (51%)	0.4

[1]Mean (SD); n (%)

[2]Wilcoxon rank sum test; NA; Pearson's Chi-squared test; Fisher's exact test

Table S2. Univariable logistic regression: HIV testing (Bonferroni selection)

Sociodemographic Factors

Variable	Term	OR (95% CI)	p-value
Age	Age	1.06 (1.04, 1.08)	<0.001
Gender (Ref. Female)	Male	1.08 (0.74, 1.55)	0.666
Residency (Ref. rural)	Urban	0.42 (0.15, 0.96)	0.0676
Marital (Ref. Divorced/widowed)	Married	0.69 (0.39, 1.25)	0.208
	Not married	0.51 (0.34, 0.73)	<0.001
Education (Ref. Bachelor and higher)	Did not complete high school	0.71 (0.41, 1.21)	0.213
	High school	1.55 (0.93, 2.49)	0.0797
	Some technical	2.46 (1.47, 4.02)	<0.001
Employment (Ref. Unemployed)	Full time	1.34 (0.92, 1.93)	0.124
	Part time	1.13 (0.77, 1.64)	0.530
Region (Ref. Astana)	Karaganda	0.79 (0.49, 1.27)	0.335
	Kostanay	2.11 (1.19, 3.86)	0.0121
	Oskemen	1.18 (0.71, 1.98)	0.517
	Petropavlovsk	0.71 (0.44, 1.14)	0.156
	Shymkent	2.24 (1.25, 3.13)	<0.001
Income level (kzt) (Ref. 100 000 or below)	200 000 and above	1.01 (0.70, 1.45)	0.957
	100 000 - 200 000	1.41 (0.99, 2.02)	0.060

Substance Use Behavior

predictor	term	OR_CI	p.value
HIV talk among users	Never	0.57 (0.41, 0.79)	<0.001
	Often	0.94 (0.58, 1.59)	0.814
Marijuana (Ref. No)	Yes	1.63 (1.21, 2.21)	<0.001
Psychostim (inj) (Ref. No)	Yes	1.53 (1.13, 2.07)	<0.001
Psychostim (inh) (Ref. No)	Yes	0.69 (0.51, 0.93)	0.015
Mix (inh or per oral) (Ref. No)	Yes	1.28 (0.95, 1.73)	0.101
Metadon (per oral) (Ref. No)	Yes	0.79 (0.59, 1.06)	0.120
Mix (inj) (Ref. No)	Yes	1.28 (0.94, 1.77)	0.128
Heroin (inj) (Ref. No)	Yes	1.14 (0.84, 1.54)	0.384
Chemsex use (Ref. No)	Yes	1.14 (0.84, 1.54)	0.391
Heroin (inh) (Ref. No)	Yes	1.10 (0.82, 1.48)	0.530
Do you think sexual activity under the influence of drugs increases the risk of sexually transmitted infections (STIs) and HIV? (Ref. No)	Yes	1.06 (0.78, 1.44)	0.712
Metadon (inj) (Ref. No)	Yes	0.99 (0.73, 1.33)	0.935
Unsafe injection use (Ref. Never)	Ever	1.43 (0.95, 2.2)	0.096
Needle use after an HIV positive person (Ref. No)	Yes	0.87 (0.64, 1.17)	0.346

Sexual Behavior

predictor	term	OR_CI	p.value
Condom use for paid sex (Ref. Never)	Always	0.75 (0.51, 1.11)	0.144
	Sometimes	0.77 (0.50, 1.18)	0.219
Paid sex for money and drugs (Ref. No)	Yes	1.42 (1.05, 1.92)	0.021

Characteristic		No<br>N = 200[1]	Yes<br>N = 1,300[1]	p-value[2]
Paid sex for food and stay (Ref. No)	Yes	1.35 (1.00, 1.83)		0.047
Anal sex with an irregular partner (past 3 months) (Ref. No)	Yes	1.28 (0.95, 1.72)		0.110
Number of sexual partners (past 3 months) (Ref. 2 or less)	3 and more	0.73 (0.53, 1.01)		0.053

Structural Access to Services

predictor	term	OR_CI	p.value
Received HIV prevention services (Ref. Yes)	No	0.44 (0.33, 0.61)	<0.001
Where do you usually go to receive a medical treatment (not related to a substance use)			
Outpatient public clinic (Ref. Yes)	No	1.24 (0.91, 1.70)	0.183
Hospital (Ref. Yes)	No	1.06 (0.79, 1.43)	0.700
Outpatient private clinic (Ref. Yes)	No	1.11 (0.83, 1.51)	0.478
HIV prevention centers (Ref. Yes)	No	0.65 (0.48, 0.89)	<0.001
NGO (Ref. Yes)	No	1.25 (0.92, 1.69)	0.153
What prevents you from always using condoms?			
Loss of control (Ref. No)	Yes	1.56 (1.14, 2.17)	<0.001

Characteristic		No<br>N = 200[1]	Yes<br>N = 1,300[1]	p-value[2]
Nothing (Ref. No)	Yes	0.70 (0.51, 0.95)		0.025
Partner refusal of condom use (Ref. No)	Yes	1.29 (0.95, 1.76)		0.112
Lack of money for a condom (Ref. No)	Yes	1.21 (0.90, 1.64)		0.202
Absence of HIV fear (Ref. No)	Yes	0.82 (0.61, 1.11)		0.205
Afraid to show fear (Ref. No)	Yes	1.18 (0.87, 1.60)		0.288
Absence of the opportunity to buy a condom (Ref. No)	Yes	1.17 (0.87, 1.58)		0.297
What prevents you from injecting drugs safely (e.g., avoiding shared needles or equipment)?				
Withdrawal symptoms (Ref. No)	Yes	0.86 (0.63, 1.17)		0.325
Nothing (Ref. No)	Yes	1.16 (0.86, 1.56)		0.329
Other (Ref. No)	Yes	1.15 (0.85, 1.54)		0.373
Loss of control (Ref. No)	Yes	0.89 (0.66, 1.22)		0.472
Absence of fear (Ref. No)	Yes	0.95 (0.71, 1.29)		0.759
Danger of being caught (Ref. No)	Yes	0.96 (0.71, 1.30)		0.782
Absence of the opportunity to buy a syringe (Ref. No)	Yes	1.04 (0.77, 1.40)		0.800
Lack of money (Ref. No)	Yes	1.01 (0.75, 1.37)		0.927

Table S3. Model Fit Statistics

Statistic	Value
N (complete cases)	1,482
AIC	1,059
McFadden R^2	0.109
AUC	0.725

Table S4. Supplementary Sensitivity Analyses

Term		OR (95%CI)	p_value
Main model + sexual behavior variables			
Age		1.10 (1.04, 1.16)	<0.01
Marital (Ref. Divorced/widowed)			
	Married	0.49 (0.11, 2.42)	0.362
	Not married	0.49 (0.14, 1.42)	0.212
Education (Ref. Bachelor and higher)			
	Did not complete high school	0.14 (0.02, 0.81)	0.034
	High school	0.66 (0.13, 2.67)	0.579
	Some technical	1.67 (0.32, 7.06)	0.503
HIV talk among users			
	Never	1.22 (0.49, 3.10)	0.669
	Often	2920469.27 (0, NA)	0.986
Psychostim (inj) (Ref. No)			
	Yes	1.30 (0.44, 4.03)	0.641
Marijuana (Ref. No)			
	Yes	2.30 (0.68, 9.60)	0.208
Received HIV prevention services (Ref. Yes)			
	No	0.93 (0.27, 3.48)	0.906
HIV prevention centers (Ref. Yes)			
	No	5.89 (1.51, 40.48)	0.027
What prevents you from injecting drugs safely? Loss of control (Ref. No)			
	Yes	1.21 (0.28, 5.38)	0.799
Paid sex for money and drugs (Ref. No)			
	Yes	1.06 (0.14, 13.10)	0.955
Paid sex for food and stay (Ref. No)			
	Yes	0.68 (0.03, 32.22)	0.828
Condom use for paid sex (Ref. Never)			
	Always	1.82 (0.15, 18.44)	0.618
	Sometimes	0.84 (0.06, 9.75)	0.887
Anal sex with an irregular partner (past 3 months) (Ref. No)			
	Yes	6.58 (1.05, 81.53)	0.077
Restricted: no HIV prevention services			
Age		1.16 (1.07, 1.28)	<0.001
Marital (Ref. Divorced/widowed)			

Term	OR (95%CI)	p_value
Married	0.14 (0.01, 1.21)	0.085
Not married	0.53 (0.06, 2.88)	0.496
Education (Ref. Bachelor and higher)		
Did not complete high school	0.05 (0.01, 0.59)	0.027
High school	0.86 (0.13, 4.84)	0.863
Some technical	2.91 (0.42, 18.64)	0.257
HIV talk among users		
Never	0.60 (0.17, 2.01)	0.420
Often	0.79 (0, 1)	1.000
Psychostim (inj) (Ref. No)		
Yes	1.57 (0.39, 7.12)	0.537
Marijuana (Ref. No)		
Yes	1.60 (0.25, 17.36)	0.650
Received HIV prevention services (Ref. Yes)		
No	13.43 (0, 192.1)	0.994
What prevents you from injecting drugs safely? Loss of control (Ref. No)		
Yes	9762.88 (0, NA)	0.997
Paid sex for money and drugs (Ref. No)		
Yes	0.17 (0.004, 5.61)	0.280
Paid sex for food and stay (Ref. No)		
Yes	44103743.70 (0, Inf)	0.999
Condom use for paid sex (Ref. Never)		
Always	18952854.18 (0, NA)	0.997
Sometimes	22524287.47 (0, NA)	0.997
Anal sex with an irregular partner (past 3 months) (Ref. No)		
Yes	2339968843.35 (0, Inf)	0.998
Restricted: no HIV prevention centers		
Age	1.08 (1.03, 1.15)	0.004
Marital (Ref. Divorced/widowed)		
Married	0.47 (0.10, 2.34)	0.341
Not married	0.55 (0.16, 1.68)	0.317
Education (Ref. Bachelor and higher)		
Did not complete high school	0.16 (0.02, 0.96)	0.053
High school	0.62 (0.11, 2.6)	0.531
Some technical	1.51 (0.28, 6.60)	0.597
HIV talk among users		
Never	0.97 (0.37, 2.52)	0.947
Often	27.07 (0, Inf)	0.989
Psychostim (inj) (Ref. No)		
Yes	1.10 (0.36, 3.56)	0.860
Marijuana (Ref. No)		
Yes	2.26 (0.66, 9.56)	0.220
Received HIV prevention services (Ref. Yes)		
No	0.93 (0.27, 3.48)	0.906
What prevents you from injecting drugs safely? Loss of control (Ref. No)		
Yes	0.77 (0.15, 3.97)	0.758
Paid sex for money and drugs (Ref. No)		
Yes	0.81 (0.10, 9.89)	0.852
Paid sex for food and stay (Ref. No)		

Term	OR (95%CI)	p_value
Yes	0.43 (0.01, 23.70)	0.651
Condom use for paid sex (Ref. Never)		
Always	2.53 (0.19, 28.66)	0.454
Sometimes	2.36 (0.14, 42.31)	0.544
Anal sex with an irregular partner (past 3 months) (Ref. No)		
Yes	25.23 (1.92, 1316.97)	0.048

This supplementary analysis evaluates whether the main findings remain robust after inclusion of sexual behavior variables and under sample restrictions designed to approximate lower service engagement and urban-only participation.
